# Measuring the Quality of Care for Older Adults With Multimorbidity: Results of the MULTIqual Project

**DOI:** 10.1093/geront/gnac013

**Published:** 2022-01-28

**Authors:** Josefine Schulze, Katharina Glassen, Nadine J Pohontsch, Eva Blozik, Tabea Eißing, Amanda Breckner, Charlotte Höflich, Anja Rakebrandt, Ingmar Schäfer, Joachim Szecsenyi, Martin Scherer, Dagmar Lühmann

**Affiliations:** Department of General Practice and Primary Care, University Medical Center Hamburg-Eppendorf, Hamburg, Germany; Department of General Practice and Health Services Research, Heidelberg University Hospital, Heidelberg, Germany; Department of General Practice and Health Services Research, Heidelberg University Hospital, Heidelberg, Germany; Institute of Primary Care, University of Zurich and University Hospital Zurich, Zurich, Switzerland; Department of General Practice and Primary Care, University Medical Center Hamburg-Eppendorf, Hamburg, Germany; Department of General Practice and Primary Care, University Medical Center Hamburg-Eppendorf, Hamburg, Germany; Department of General Practice and Primary Care, University Medical Center Hamburg-Eppendorf, Hamburg, Germany; Department of General Practice and Primary Care, University Medical Center Hamburg-Eppendorf, Hamburg, Germany; Department of General Practice and Primary Care, University Medical Center Hamburg-Eppendorf, Hamburg, Germany; Department of General Practice and Health Services Research, Heidelberg University Hospital, Heidelberg, Germany; Department of General Practice and Primary Care, University Medical Center Hamburg-Eppendorf, Hamburg, Germany; Department of General Practice and Primary Care, University Medical Center Hamburg-Eppendorf, Hamburg, Germany

**Keywords:** Chronic care, Comorbidity, Patient-centered care, Primary care, Quality standard

## Abstract

**Background and Objectives:**

Providing health care for older adults with multimorbidity is often complex, challenging, and prone to fragmentation. Although clinical decision making should take into account treatment interactions, individual burden, and resources, current approaches to assessing quality of care mostly rely on indicators for single conditions. The aim of this project was to develop a set of generic quality indicators for the management of patients aged 65 and older with multimorbidity that can be used in both health care research and clinical practice.

**Research Design and Methods:**

Based on the findings of a systematic literature review and eight focus groups with patients with multimorbidity and their family members, we developed candidate indicators. Identified aspects of quality were mapped to core domains of health care to obtain a guiding framework for quality-of-care assessment. Using nominal group technique, indicators were rated by a multidisciplinary expert panel (*n* = 23) following standardized criteria.

**Results:**

We derived 47 candidate quality indicators from the literature and 4 additional indicators from the results of the focus groups. The expert panel selected a set of 25 indicators, which can be assigned to the levels of patient factors, patient–provider communication, and context and organizational structures of the conceptual framework.

**Discussion and Implications:**

We developed a comprehensive indicator set for the management of multimorbidity that can help to highlight areas with potential for improving the quality of care and support application of multimorbidity guidelines. Furthermore, this study may serve as a blueprint for participatory designs in the development of quality indicators.

With demographic aging and the rise of chronic conditions, caring for patients with multimorbidity has become a significant challenge across all health care settings (Afshar et al., 2015; Kingston et al., 2018; Uijen & van de Lisdonk, 2008). In contrast to comorbidity, which is the combination of an index condition of primary interest with additional conditions (Feinstein, 1970), multimorbidity refers to the joint presence of multiple, potentially interacting chronic health conditions, “where one is not necessarily more central than the others” (Boyd & Fortin, 2010). Multimorbidity is linked to increased health care utilization and costs (Bähler et al., 2015; Glynn et al., 2011), and patients frequently report functional limitations, psychological distress, and reduced quality of life (Fortin et al., 2006; Jindai et al., 2016; Williams & Egede, 2016). In primary care practices, patients with multiple chronic conditions account for more than half of all consultations (Cassell et al., 2018). Although multimorbidity is the rule rather than the exception in older adults (Salive, 2013; Violan et al., 2014), traditional clinical practice guidelines often focus on the management of single diseases with little consideration of comorbidities (Hughes et al., 2013; Tinetti et al., 2004). Primary care providers consider the inadequacy of disease-oriented guidelines and disintegration of care as major obstacles in providing optimal care for persons with multimorbidity (Sinnott et al., 2013). Uncoordinated and fragmented care increases the risk of greater treatment burden, polypharmacy, poor adherence, and can lead to potentially harmful treatment interactions, especially in the presence of discordant conditions with competing health care requirements (Lorgunpai et al., 2014; Wallace et al., 2015).

To date, there is no consensus on the metrics that best reflect the quality of health care delivered to patients with multimorbidity. Recent studies have targeted the question of which outcome measures are most relevant to address the impact of health care interventions for adults with multimorbidity (Hurst et al., 2020; Smith et al., 2018). Quality of care for people with multimorbidity is often evaluated by aggregating performance measures for single conditions (Valderas et al., 2019). However, previous research suggests that this strategy might lead to worse results in quality assessment when discordant conditions are present (Ricci-Cabello et al., 2015). Ferris et al. (2017) identified the application of condition-specific performance measures as an important risk factor for fragmented and burdensome care. Implementing generic quality metrics that correspond to patients’ priorities offers an opportunity to improve health care substantially. Quality indicators reflect processes, structures, and outcomes sensitive to quality improvement and may provide a quantitative basis for the assessment of clinical performance (Mainz, 2003). There is consensus that for older adults with multimorbidity the focus of quality assessment should be directed at primary care, as this setting is best suited to meet the needs of this patient group for comprehensive and patient-centered care as well as continuity and coordination (Moffat & Mercer, 2015; World Health Organization & United Nations Children’s Fund, 2018). In Germany, the first point of contact for older adults with chronic conditions is usually the general practitioner (GP). However, there is no legally binding gatekeeper system. Rather, the GP’s coordinating and gatekeeping role is based on trust and a long-standing relationship with the patient. This, in turn, can only endure if conditions and their consequences for everyday life are addressed in a way that is aligned with patient preferences.

The aim of our study was to identify indicators and guideline recommendations (as a basis for indicators) with relevance to multimorbidity care in Germany and amend these findings with quality aspects meaningful to patients with multimorbidity. Our study proposes a conceptual framework for quality of care and a set of quality indicators for the management of older adults (aged 65 and older) with multimorbidity in primary care based on a systematic consensus approach. Although the importance of patient involvement in indicator development is well appreciated, a gold standard on effective engagement strategies is still lacking (Kötter et al., 2013). To ensure the representation of quality aspects relevant to the target group, patients were involved at multiple stages of the process.

## Method

The MULTIqual project implemented a mixed-methods approach to combine the best available evidence on the effectiveness of interventions with clinical expertise on the management of patients with multimorbidity. We derived candidate quality indicators based on a systematic literature review. To inform the decision-making process and amend the literature-based indicator set, focus groups with patients affected by multimorbidity and their family members were conducted. We convened a multidisciplinary expert panel that further refined and selected a preliminary indicator set via nominal group technique (McMillan et al., 2016). In addition, we developed a conceptual model that defines core components of health care delivery for this target group. The methodology and results of indicator development are reported following the standards for guideline-based performance measures established by the Guidelines International Network (GIN) Performance Measures Working Group (Nothacker et al., 2016).

### Systematic Literature Review

A systematic literature review was performed to identify existing references on clinical guidance and quality metrics for multimorbidity care in the electronic databases PubMed, CINAHL, CareLit, Cochrane Library, PsycInfo, Livivo, and GeroLit over a period of 10 years (from 2007 to September 2017) as well as GIN and National Guideline Clearinghouse databases. The full search strategy is provided in Supplementary Table 1. We also searched via OpenGrey, HSRProj, and ICTRP databases to identify ongoing or unpublished research and conducted a manual search based on the reference lists of all publications selected for the final review. References were included if they reported a methodologically rigorous development process. Publications limited to the management of polypharmacy or specific index conditions with respect to comorbidities were excluded. We did not define any restrictions regarding clinical settings or operationalization of multimorbidity. Based on title and abstract screening, papers were selected for full-text reading if they were available in English or German. Eligible references were reviewed for inclusion by two researchers independently (J. Schulze, T. Eißing) and discussed with a third reviewer (D. Lühmann) in order to reach a consensus. The methodological quality of references providing clinical guidance was assessed using the AGREE II instrument (AGREE Next Steps Consortium, 2014). We extracted all recommendations and quality metrics directed at the clinical management of people with multimorbidity and made suggestions for candidate quality indicators. Taking into consideration that high-quality evidence in the field of health care for patients with multimorbidity is scarce (Smith et al., 2021), we chose to follow the best evidence approach. Wherever possible, quality indicators were derived based on the recommendation with the highest possible level of evidence. In cases where sources with lower levels of evidence provided aspects not yet covered, these recommendations were extracted as well.

### Focus Groups

We invited randomly selected GPs from the northern and southern regions of Germany (Hamburg and Heidelberg and surroundings) to support the study by recruiting patients aged 65 and older with three or more chronic conditions from their practice. Patients were encouraged to invite family members to participate in the study as well. We convened eight focus groups with patients with multimorbidity and three focus groups with patients’ family members in December 2018 and January 2019. Informed written consent was obtained from all participants prior to the beginning of the focus groups. Discussions in the focus groups followed a semistructured format.

Due to its complexity and multifaceted nature, the term “quality” is often understood differently by laypersons. Therefore, we did not address this term directly in the focus group guide, but approached it indirectly by asking questions about positive and negative experiences with care (Sofaer & Firminger, 2005), changes in health care needs when living with multiple conditions, and suggestions for quality improvement. Digital recordings were transcribed verbatim. In line with Sandelowski (2000), we used a descriptive qualitative methodology to uncover important issues for patients and relatives. We followed Kuckartz’s (2012) approach to qualitative content analysis to analyze the collected data in a systematic but flexible way. As part of the coding process, we developed inductive and deductive codes using MAXQDA software: Emerging quality aspects were matched to the previously identified literature-based indicators (deductive codes). The working group derived new quality indicators when aspects of relevance to patients or family members were not represented in the literature (inductive codes). Each category was described in a code memo to help allocation of text passages to categories (an example is given in Supplementary Table 2). Further details can be found in a separate publication (Pohontsch et al., 2021).

### Expert Panel

An independent interdisciplinary expert panel was convened to comment, rate, and select candidate quality indicators via nominal group technique that included an online rating and a face-to-face meeting. To reflect the broad range of possible care constellations and care pathways in multimorbidity, the panel included the most central stakeholders from a variety of clinical fields (general practice, geriatrics, nursing, social work, physical therapy, and pharmacology), health economy as well as researchers with methodological expertise in quality and health services research and patient representatives. Candidates were invited based on their clinical or methodological expertise. We contacted patient organizations to recruit patient representatives with lived experience of chronic conditions who were able to participate as advocates for the interests of people with multimorbidity. To minimize bias, we ensured a gender-balanced representation within the panel, recruited experts from different regions of Germany, and reviewed conflicts of interest disclosed by all potential panel members.

Prior to the first stage of the consensus process, participants received a handbook containing basic information on the purpose of the study as well as information on terminology and methodology of indicator development. Challenges related to indicator development in the field of multimorbidity, methodological questions, and the role of the experts were explained and discussed in preparatory video conferences with up to 10 panel members each. An online rating was conducted from November 2018 to January 2019 via EFS Survey (Questback). Panel members were encouraged to comment on the candidate indicators to clarify descriptions and data sources. Rating criteria (Table 1) were based on the QUALIFY tool (Reiter et al., 2008) following the German National Disease Management Guidelines manual on quality indicators (Altenhofen et al., 2009).

**Table 1.** Rating Criteria for Candidate Quality Indicators

Category	Key statement	Response format
Significance	“The indicator covers essential aspects of quality of life, morbidity, or mortality, or relevant care processes or structures.”	Four-level Likert scale: 1 = strongly disagree 2 = disagree 3 = agree 4 = strongly agree
Clarity of definition	“The indicator is defined clearly and unambiguously.”	
Possibility to influence the indicator manifestation	“The indicator refers to aspects of care that are under the practitioner’s control.”	
Strength of evidence	“The existence of the measured structure/process leads to a better result” or “The measured outcome is associated with a higher quality of care.”	
Potential risks/undesirable effects	“Does the indicator create potential misincentives?”	Dichotomous (Yes/No)

The results of the first stage of the consensus process were analyzed by assessing the proportion of “agree” and “strongly agree” votes for the dimensions significance, strength of evidence, possibility to influence the indicator manifestation, and clarity of definition. Consistent with established methods for guideline and indicator development in Germany (Schorr et al., 2017), an agreement of at least 75% in all categories was regarded as the general acceptance of an indicator. Less than 75% agreement in more than two of the four categories suggested rejection. Mixed ratings of indicators were deemed inconclusive and consequently assigned to open discussion. Votes on potential risks and undesirable effects were evaluated separately and furthermore, the panel was asked for free-text comments for indicator improvement. The results of the first stage were then made available to the panel in aggregated form. The second stage took place in February 2019 as an in-person meeting with all panel members for discussion, refinement where necessary, and selection of the preliminary set of quality indicators via open voting.

### Measurement Framework

Based on our findings, we inductively derived a conceptual framework to capture the most vital aspects of quality of care for this target group. In a first step, all extracted recommendations from the literature and focus groups were categorized into different domains of care (Agency for Healthcare Research and Quality [AHRQ], 2014). In a second step, these care domains were mapped onto the levels of interventions that affect health care delivery (Taplin et al., 2012) to illustrate the relationships between the targeted aspects of care. Discrepancies were discussed within the working group until consensus was reached.

## Results

**Figure 1.**
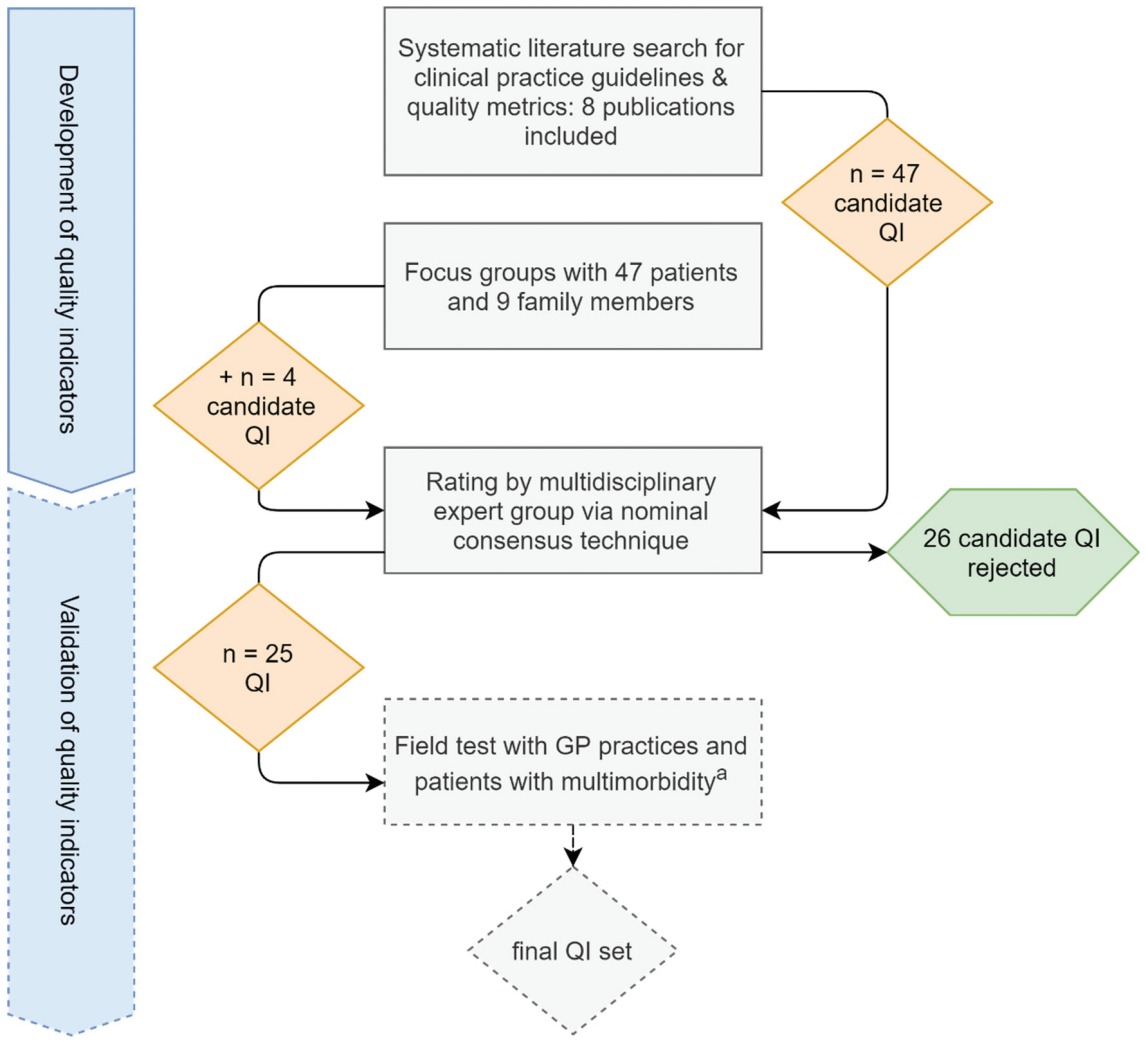
Flowchart of indicator development and number of resulting quality indicators (QIs). ^a^Further details to be published separately (Schulze et al., 2022).

Figure 1 illustrates the results of the different methodological approaches.

### Literature Review

**Figure 2.**
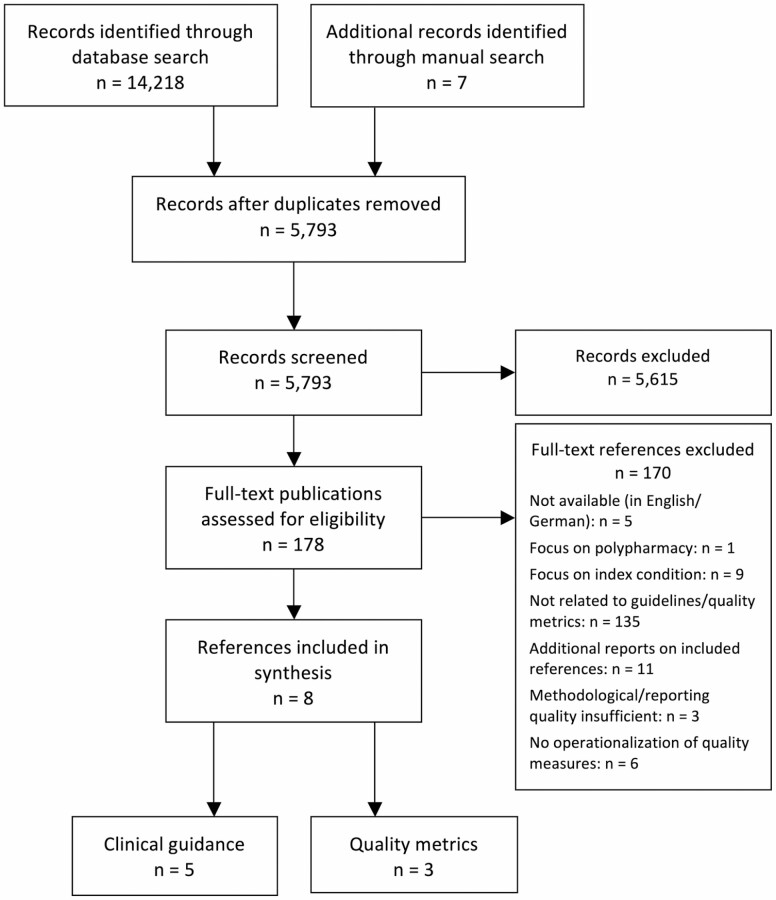
Flowchart of systematic literature review.

About 14,218 references were identified through database searching and seven additional papers through hand searching. After removal of duplicates, titles and abstracts of 5,793 hits were screened and 178 papers were retained for full-text review (Figure 2). In total, three guidelines and two guidance papers as well as three references on quality metrics were included in the final review. Table 2 lists all included documents.

**Table 2.** Characteristics of Included References

Reference	Country of origin	Document type	Target population	Target setting	Target audience
National Institute for Health and Care Excellence (NICE), 2016	UK	Guideline	Adults with two or more long-term health conditions, not including people with only mental and not physical health problems	Primary and secondary care	Patients, health care professionals (primarily GPs, geriatricians, specialists)
German College of General Practitioners and Family Physicians, 2017	Germany	Guideline	Adults with three or more chronic conditions	Primary care	Patients, primary care practitioners
American Geriatrics Society Expert Panel on the Care of Older Adults with Multimorbidity, 2012	USA	Guiding principles	Older adults with multiple chronic conditions	Primary and secondary care	Clinicians, researchers, public health professionals, payers, policymakers, interested public
Muth et al., 2014	North America and Europe	Guiding principles	Patients with multiple chronic conditions	Decision making in primary care	Family physicians, primary care researchers, medical education, training services
Palmer et al., 2018	EU	Health care framework	Patients with multiple chronic diseases or conditions	European health care systems	National policymakers, providers
NICE, 2017	UK	Quality standard	Adults with two or more long-term health conditions	Primary and secondary care	Providers, health care practitioners, commissioners, adults with multimorbidity
National Quality Forum, 2012	USA	Guiding principles and high-priority domains for quality measurement	Adults with two or more conditions that collectively have an adverse effect on health status, function, or quality of life and that require complex health care management, decision making, or coordination	Various health care settings	Providers, health professionals, purchasers, health plans, consumers, researchers
Working Group on Health Outcomes for Older Persons with Multiple Chronic Conditions, 2012	USA	Standard health outcome measures	Older adults with multiple chronic conditions	Various health care and residential settings	Clinical researchers, policymakers, practitioners

Results of the quality appraisal were overall positive for two of the three guidelines (see Supplementary Figure 1 for AGREE II ratings). We extracted 81 recommendations and six performance measures from the literature. We did not derive quality indicators based on recommendations that were not applicable to the German health care system or addressed rather specific aspects of care, for example, detailed requirements for the assessment of frailty. All recommendations were translated into quality indicators by defining numerators, denominators, and data sources. To provide users with a systematic but easy-to-perform computation, we chose to operationalize multimorbidity as the presence of three or more chronic conditions. Previous studies propose using the more selective threshold of ≥3 conditions to identify patients with multimorbidity (Lee et al., 2021; van den Bussche et al., 2011), as the often-used criterion of ≥2 conditions (Johnston et al., 2019) results in prevalence rates of 75%–99% in primary care for this age group (Cassell et al., 2018; Fortin et al., 2005). There was a substantial overlap within care domains, resulting in a list of 47 candidate quality indicators. For example, both the National Institute for Health and Care Excellence (NICE) guideline (2016) and quality standards (2017) suggest reviewing medications for risks and benefits, as well as potential interactions, to determine whether any should be discontinued or changed. The DEGAM guideline (2017) points out that a higher number of different medications increases the risk of drug interactions, adverse effects, and nonadherence.

### Focus Groups

A total of 47 patients and nine family members (spouses and adult children) took part in the focus groups. A large proportion of literature-based quality indicators were supported by the aspects suggested in the focus groups. For instance, the aspect of reviewing medication was also raised in the focus groups: “Especially in the case of multiple chronic conditions, I find it extremely important that the GP explains to me that the drugs are compatible, that there is no interaction or that it is possible that they will cancel each other out or even worsen the condition, as in the case of my mother […]” (focus group with family members, cited from Pohontsch et al., 2021). Four additional quality aspects emerged from the discussions and were added to the list of candidate indicators: offer self-management support and education, regular updates of medication plans, periodic checkups, and GP-coordinated care. Participants considered information on education and self-management strategies to be highly relevant to quality of care. In the focus groups, participants emphasized the value of up-to-date medication plans for patient safety, especially when there are many medications or multiple prescribing physicians. They advocated for GPs to take on a coordinating role, including targeted referrals to a network of specialized treatment providers. Participants shared their preference for regular screenings and checkups to detect potential health deterioration at an early stage. These findings were presented to the expert panel and evaluated, discussed, further operationalized, and selected in the nominal group process (Pohontsch et al., 2021).

### Expert Panel

The expert panel consisted of 23 experts (as listed in the Acknowledgments section), with a drop out of *n* = 4 in the second stage due to time constraints. After the first stage, 23 indicators met the criteria for general acceptance, whereas 22 showed mixed ratings and six were rejected. In these cases, rejection was due to concerns about the evidence base and the link between indicator scores and better outcomes. The working group assessed all free-text comments for relevance and applicability: Some comments were incorporated into indicator descriptions, reference periods, or data collection. Relevant comments that could not be applied directly were assigned to open discussion. Out of the indicators with mixed ratings, seven were accepted (after refinements) in the second stage of the consensus process. However, after discussion, the panel decided to drop five indicators with positive ratings in the first round because of overlaps with other indicators that were found to be more appropriate and clearer in operationalization. Rejected candidate indicators are listed in Supplementary Table 3. Ultimately, 25 quality indicators were included in the final set. The indicator on reviewing medication, for example, obtained over 90% agreement in all categories and was retained in the final set by consensus. Table 3 provides an overview of the accepted indicators.

**Table 3.** Description of Quality Indicators Accepted by Expert Panel

Quality indicator	Numerator	Denominator	Data source
**Screening for depression**	No. of patients whose risk of depression was assessed using screening questions	No of patients (65+) with ≥3 cc without a prior diagnosis of depression	GP survey/medical records
**Proactive pain assessment**	No. of patients who were asked about the presence of pain	No. of patients (65+) with ≥3 cc	GP survey/medical records
**Monitoring of pain management**	No. of patients with chronic pain whose pain management was monitored and adjusted if necessary	No. of patients (65+) with ≥3 cc	GP survey/medical records
**Addressing financial support needs**	No. of patients who were asked about their need for financial support	No. of patients (65+) with ≥3 cc	GP survey/medical records
**Quality of life assessment**	No. of patients who had a discussion about their subjective quality of life	No. of patients (65+) with ≥3 cc	GP survey/medical records
**Assessment of symptom burden**	No. of patients whose symptom burden was assessed using validated measurement tools	No. of patients (65+) with ≥3 cc	GP survey/medical records
**Assessment of biopsychosocial support needs**	No. of patients whose biopsychosocial support needs were assessed and documented according to ICF	No. of patients (65+) with ≥3 cc	GP survey/medical records
**Eliciting patient preferences**	No. of patients whose priorities, goals, and values were discussed and documented	No. of patients (65+) with ≥3 cc	GP survey/medical records
**Involving partners, family, and caregivers**	No. of patients who had a discussion whether and to what extent partners, family, and caregivers should be involved in important decisions	No. of patients (65+) with ≥3 cc	GP survey/medical records
**Patient education/self-management**	No. of patients who were offered participation in a patient training or support group or given a written self-management plan	No. of patients (65+) with ≥3 cc	Patient survey
**Identification of patients with multimorbidity**	No. of patients for whom the presence of multimorbidity was identified and labeled in their file	No. of patients (65+) with ≥3 cc	GP survey/medical records
**Information about medication**	No. of patients who were informed about their medication (indication, effect, intake)	No. of patients (65+) with ≥3 cc receiving pharmacological treatment	GP survey/medical records
**Information about potential benefits and harms of treatment options**	No. of patients who were informed about potential benefits and risks of treatment options prior to treatment decisions	No. of patients (65+) with ≥3 cc	GP survey/medical records
**Shared decision making**	No. of patients who state that they are involved in treatment decisions to the extent they wish	No. of patients (65+) with ≥3 cc	Patient survey
**Mutual agreement on treatment goals**	No. of patients with whom treatment goals were established	No. of patients (65+) with ≥3 cc	GP survey/medical records
**Written treatment plan**	No. of patients with a written treatment plan	No. of patients (65+) with ≥3 cc	GP survey/medical records
**Medication review**	No. of patients who received a review of their medication	No. of patients (65+) with ≥3 cc with long-term medication	GP survey/medical records
**Regular updates of medication plan**	No. of patients whose medication plan was checked for updates in the last 3 months	No. of patients (65+) with ≥3 cc with ≥3 long-term medications	GP survey/medical records
**Monitoring adherence to treatment**	No. of patients whose adherence to treatment was assessed	No. of patients (65+) with ≥3 cc	GP survey/medical records
**Assessment of treatment burden**	No. of patients who had a discussion of their treatment burden	No. of patients (65+) with ≥3 cc	GP survey/medical records
**Assigning responsibility for coordination of care**	No. of patients with whom it was agreed and recorded which health care provider is responsible for the overall coordination of care	No. of patients (65+) with ≥3 cc	GP survey/medical records
**Comprehensive care documentation**	No. of patients for whom reports from all health care providers involved are accessible to the care coordinator	No. of patients (65+) with ≥3 cc	GP survey/medical records
**Documentation of adverse drug reactions**	No. of included practices/units where the identification and documentation of adverse drug reactions follow a standardized procedure	No. of included practices/units	Practice survey
**Training programs addressing the management of ** patients** with multimorbidity**	No. of practices/units where (a) at least one physician and (b) at least one member of the nonphysician staff have participated in training programs for multimorbidity	No. of included practices/units	Practice survey

*Note:* No. of patients = number of patients; cc = chronic conditions; GP = general practitioner; ICF = International Classification of Functioning, Disability, and Health.

### Measurement Framework

**Figure 3.**
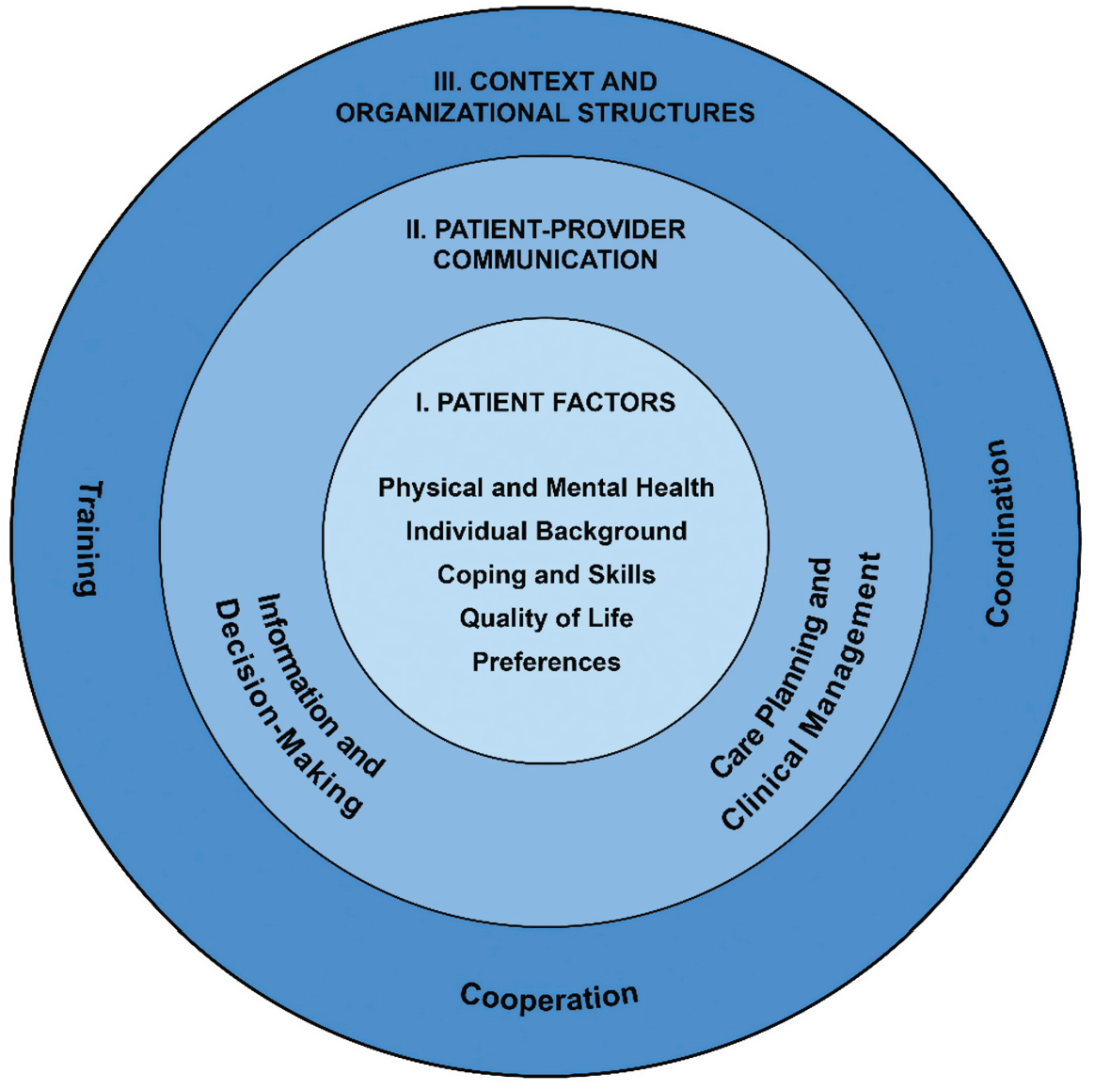
Conceptual framework of quality of health care for older adults with multimorbidity. *Note:* A guiding framework for categorizing quality indicators in relation to domains of care and different levels of influences that affect health care, adapted from Taplin et al. (2012).

Resulting from this iterative process, we developed a conceptual framework for quality-of-care assessment in older adults with multimorbidity (Figure 3). For this purpose, we derived 13 care domains from included references and focus group data, such as training or decision making. Identified care domains were then categorized into three target levels: (a) patient factors, (b) patient–provider communication, and (c) context and organizational structures. By using this model as a guiding framework, we wanted to ensure that the final indicator set covered the core components of quality of care. The proposed framework can be used to illustrate and structure quality measurement for this target group, indicating for which areas of interest quality metrics are available.

## Discussion

Here we presented the first thoroughly developed indicator set for multimorbidity for use in the German health care system. Through systematic literature review, eight relevant publications were identified, and 81 recommendations and six quality metrics were extracted and operationalized for quality measurement. Four additional quality indicators were derived based on the findings from 11 focus groups. The resulting 51 candidate quality indicators were evaluated by an independent multidisciplinary expert panel that selected a set of 25 quality indicators via a two-stage nominal group technique. Based on the obtained results, we developed a measurement framework that conceptualizes and structures quality measurement for older adults with multimorbidity.

When reviewing the scientific literature, we encountered significant challenges related to the lack of high-quality evidence in this area (Smith et al., 2021). In our set, only a few indicators are based on the results of randomized controlled trials, whereas the majority is supported by lower levels of evidence. We addressed this limitation by integrating perspectives of patients, practitioners, and researchers to supplement systematic evidence with clinical expertise and lived experience and achieve greater acceptance among these user groups. In combining different approaches for patient involvement, we demonstrated the feasibility of a multilevel approach to patient involvement. Using focus groups, we were able to capture additional quality aspects not reflected in previous research. This project demonstrates how qualitative studies of patients’ and carers’ views on care pathways and (unmet) care needs can enrich traditional processes of indicator development. Another major strength of our study is the diversity of professional backgrounds within the expert panel. However, panel members were invited to participate based on their expertise and were not appointed as official representatives of stakeholder organizations.

The indicator set has only limited applicability to routine quality assessment. Because most recommendations in multimorbidity guidelines refer to communication and decision-making processes between providers and patients, the indicators address a broad spectrum of “soft” factors not captured in routine data. The lack of standardization of clinical documentation in ambulatory care poses an additional challenge for both research and quality management. Data collection requirements vary widely for medical practices, with more than 140 different practice management systems currently in use (National Association of Statutory Health Insurance Physicians, 2020). Nonetheless, our findings offer directions for future implementation in electronic documentation systems and can be adopted once documentation standards have been picked up. In addition, patient-reported experience data can be integrated into patient surveys, which are already a part of the legally required quality management for practices in Germany (Federal Joint Committee [GBA], 2020).

A distinct feature of our indicator set in comparison to condition-specific quality measures is the particular focus on processes of care. As older patients with multimorbidity are a heterogeneous target group, standardized health outcomes are difficult to compare. While adverse events, health-related quality of life, and functioning have previously been established as relevant quality domains (Valderas et al., 2019), studies have shown associations between those variables and different patterns of multimorbidity (Marengoni et al., 2020; Panagioti et al., 2015; Sum et al., 2019). Outcome parameters may be influenced by several factors beyond the control of practitioners, and some aspects of health care aspects will only show their impact on health outcomes within a longer time frame. In comparison, process indicators are more sensitive to changes in care (Mant, 2001) and less prone to differences in case-mix (AHRQ, 2015).

Our findings are in many ways consistent with the NICE quality standards for multimorbidity (2017). Both sets took a generic approach to define quality of care in multimorbidity and share their mutual goal to describe measures sensitive to quality improvement, which applies to care processes as well as structures and outcomes. Vital aspects of patient-centered clinical management are reflected within both sets: defining responsibility for care coordination, discussing priorities and goals, and reviewing medication as well as other treatments. We also found similarities between the quality dimensions obtained in our study and an integrated model of patient-centeredness recently adapted for this target group (Kivelitz et al., 2021). Moreover, our framework aligns quality measurement with an approach to personalized clinical management based on patient preferences and shared decision making. Another person-centered measurement framework for people with multimorbidity was developed by the National Quality Forum (NQF, 2012). Unlike our project, their objective was to provide guidance for public reporting and performance-based payment programs. Therefore, the NQF framework also includes different providers and types of care that are not relevant to the user perspective of our project. A shared feature is that patient preferences are at the centerpiece of both conceptual frameworks. Likewise, the included aspects of quality relevant to primary care show great similarities. Two aspects missing from our set are advance care planning and health literacy. Both were covered in candidate indicators but were ultimately dropped in the second stage of the consensus process. During the discussion, it became clear that although both aspects were considered relevant, there was a lack of clarity in operationalization and insufficient evidence to apply these indicators to the entire target group. It remained unclear which minimum requirements should be placed upon these processes and their documentation in order to provide a reliable operationalization. Outcomes such as advance health care directives in place were not considered equally relevant for all patients of the target group. Similarly, there was no majority on how to assess health literacy without imposing disproportionate burden on primary care practices. These considerations highlight the importance of further research in those areas and corresponding updates to the indicator set. A universally accepted approach to conceptualizing and operationalizing multimorbidity is still lacking (Johnston et al., 2019). Although our operationalization of multimorbidity as the presence of at least three chronic conditions clearly brings advantages in terms of practicality, there is reasonable doubt that the number of diagnoses can be regarded as a reliable unit of information. Application of these indicators in a primary care sample could yield further insights into effective identification of multimorbidity.

While developing indicators is essential to identify and control aspects relevant for quality improvement, their clinical utility strongly depends on their feasibility and their capability to indicate differences across providers (Campbell et al., 2002). As a next step, we conduct a field test in a sample of 35 GP practices and 350 older patients with multimorbidity to assess clinimetric properties of the indicators. Continued evaluation and refinement will be essential to adjust metrics across care settings and to determine which measures add value to clinical practice (AHRQ, 2011).

## Conclusions and Implications

This study described the development of a comprehensive set of 25 indicators to assess health care quality for multimorbidity. We adopted an innovative approach, bringing together an established methodology for indicator development and active patient involvement. With these quality indicators, we aim to provide health professionals, researchers, policymakers, and educators in the field of aging with a building block for managing multimorbidity in the face of changing demographics. The indicator set can be used for quality monitoring in primary care as well as health care research and will be proposed as an additive element for the German multimorbidity guideline. The indicators are an alternative to the inadequate combination of disease-focused quality metrics and can be used to promote patient-centered care for this target group. They will help to elicit approaches to quality improvement and advance standardization of care delivery. Nevertheless, their implementation should be continuously evaluated in order to promote dynamic maintenance and adaptation of the indicators.

## Supplementary Material

gnac013_suppl_Supplementary_Material
