# Supplementary material for: Measuring the Quality of Care for Older Adults With Multimorbidity: Results of the MULTIqual Project
Source: Gerontologist. 2022 Jan 28;62(8):1135–46. doi: 10.1093/geront/gnac013 (PMC9451020; doi:10.1093/geront/gnac013)
Supplement: gnac013_suppl_Supplementary_Material [file gnac013_suppl_supplementary_material.docx]

*The Gerontologist* Online Supplementary Material: Schulze, Josefine; Glassen, Katharina; Pohontsch, Nadine Janis; Blozik, Eva; Eißing, Tabea; Höflich, Charlotte; Breckner, Amanda; Rakebrandt, Anja; Schäfer, Ingmar; Szecsenyi, Joachim; Scherer, Martin; & Luehmann, Dagmar. Measuring the quality of care for older adults with multimorbidity: Results of the MULTIqual project.

**Supplementary Table 1.** Search strategy for the systematic literature search

| Components | Specification | | |
| --- | --- | --- | --- |
| Search terms (English) | multimorbid*  multiple chronic  complex patient  complex chronic  polymorbid*  multi-condition  polypath*  multiple long-term | quality indicator  quality of care  care quality  quality of treatment  treatment quality  quality of medical care  predictor of quality  quality standard  assessment of quality  quality assessment  measuring quality  performance measures  measures of quality  quality measures  system performance  clinical indicators  review criteria  quality improvement  clinical audit  indicator development  develop* indicators | guideline  guidance  recommendations for practice  practic* recommendations  evidence-based recommendations  guiding principle  concept of care  standards  clinical protocol  critical pathway |
| Keyword register | "Guideline"[Publication Type]  "Guidelines as Topic"[Mesh]  "Guideline Adherence"[Mesh]  "Quality Control"[Mesh]  "Quality Improvement"[Mesh]  "Quality Indicators, Health Care"[Mesh] | | |
| Electronic databases | Pubmed, CINAHL, Carelit, PsycInfo, Livivo, GeroLit, Cochrane Library, Guidelines International Network (G-I-N), National Guideline Clearinghouse (NGC), OpenGrey, HSRProj, ICTRP | | |
| Websites of relevant institutions | AHQR, AWMF, aQua (AQUIK, QISA), ÄZQ, AQUIK, NICE, IQWiG, IQTIG | | |
| Search period | 2007 to September 2017 (query date) | | |
| Languages | English, German | | |
| Implementation of search strategy | Use of English search terms in all databases using the keyword register, German search terms in German-language databases, single keyword search in guideline/indicator databases, search on websites of relevant institutions, hand search | | |

**Supplementary Figure 1.** Quality appraisal of included guidelines using AGREE II

**Supplementary Table 2.** Example of inductive coding procedure - excerpt from the coding manual

| Code | Description | Quote |
| --- | --- | --- |
| GP-coordinated care | Text passages indicating that patients prefer their care to be coordinated by their GP | *“[...] when the diagnoses are made and the GPs know what's going on, then they should actually act as a control centre and have the possibility to coordinate everything.” (cited from Pohontsch et al., 2021)* |

**Supplementary Table 3.** Quality indicators rejected by the expert panel

| Quality Indicator | Numerator | Denominator | Reason for rejection |
| --- | --- | --- | --- |
| Screening for anxiety disorders | No of pts whose risk of anxiety disorders was assessed with screening questions | No of pts aged 65 and over with three or more chronic conditions without prior diagnosis of anxiety disorder | Anxiety disorders as a heterogeneous group, limited data on the relationship between multimorbidity and anxiety |
| Comprehensive pain assessment | No of pts who received a comprehensive pain assessment | No of pts aged 65 and over with three or more chronic conditions with chronic pain | Minimum requirements unclear, preference is given to other indicators on pain assessment and management |
| Screening for frailty | No of pts for which the presence of frailty was assessed with valid instruments | No of pts aged 65 and over with three or more chronic conditions | Relationship between multimorbidity and frailty is not yet well understood |
| Information on support services | No of pts who were informed about support services | No of pts aged 65 and over with three or more chronic conditions with complex needs | Definition too broad with risks for validity and reliability |
| Assessment of living situation | No of pts whose living situation was assessed | No of pts aged 65 and over with three or more chronic conditions | Rejected in 1^st^ round; lack of clarity, addresses the interface between social work and the medical field |
| Information on housing adaptation | No of pts who were informed about possibilities for housing adaptation | No of pts aged 65 and over with three or more chronic conditions | Rejected in 1^st^ round; lack of clarity, addresses the interface between social work and the medical field |
| Eliciting social support | No of pts with a discussion of social support strategies | No of pts aged 65 and over with three or more chronic conditions | Vagueness in operationalization; overlap with indicator “Assessment of biopsychosocial support needs” |
| Eliciting health literacy | No of pts whose health literacy was assessed and taken into account in care planning | No of pts aged 65 and over with three or more chronic conditions | Operationalization unclear; lack of evidence on link between assessment and action as well as clinical utility of short screening instruments |
| Eliciting coping strategies | No of pts with a discussion of coping strategies | No of pts aged 65 and over with three or more chronic conditions | Lack of clarity |
| Eliciting subjective impairment | No of pts with a discussion of subjective impairment due to chronic conditions | No of pts aged 65 and over with three or more chronic conditions | Overlap with indicators “Assessment of symptom burden” and “Quality of life assessment” that are operationalized more clearly |
| Advance care planning | No of pts with a discussion of advance care plans | No of pts aged 65 and over with three or more chronic conditions | Operationalization and minimum requirements unclear; overlap with indicator “Eliciting patient preferences”; Applicability to entire target population questionable |
| Evaluation of goal attainment | No of pts with regular evaluation of goal attainment | No of pts aged 65 and over with three or more chronic conditions | Lack of clarity, definition too broad |
| Elevated treatment burden | No of pts who state that they feel overwhelmed by their health-related tasks | No of pts aged 65 and over with three or more chronic conditions | Would support a deficient view of patients' resources and situations |
| Good adherence to treatment | No of pts who state that they adhere to their treatment plan | No of pts aged 65 and over with three or more chronic conditions | Overlap with the process indicator “Assessment of treatment burden”, which has a stronger link to the therapeutic alliance |
| Checking side effects | No of pts who state that they are being asked about harms from medication | No of pts aged 65 and over with three or more chronic conditions with long-term medication (medication with an intake period of at least 6 months) | Limited reliability of data collection |
| Brown bag review | No of pts who received a brown bag review | No of pts aged 65 and over with three or more chronic conditions with long-term medication | Overlap with indicators “Medication review“ and “Regular updates of medication plan” that are more in line with current practice |
| Discussion of prognosis | No of pts who were offered a discussion of prognosis | No of pts aged 65 and over with three or more chronic conditions | Unclear whether desired by patients; offer should be made sensitively without false incentives |
| Incorporating patient preferences | No of pts who state that their preferences are taken into account in the decision-making process | No of pts aged 65 and over with three or more chronic conditions | Lack of clarity and minimum requirements |
| Network of involved health professionals | No of pts whose primary care provider can list all health professionals involved in their care | No of pts aged 65 and over with three or more chronic conditions | Completeness of list difficult to verify |
| Interprofessional communication | No of pts for which the health professions involved coordinate diagnostics and therapy | No of pts aged 65 and over with three or more chronic conditions with care plans that involve multiple professions | Lack of clarity and minimum requirements |
| Flexible consultation length | Providers that enable older patients with multimorbidity to have consultations according to their needs | Number of included practices/units | Rejected in 1^st^ round; unclear relationship between actual length of consultation and quality |
| Satisfaction with consultation length | No of pts who state that they are satisfied with the consultation length | No of pts aged 65 and over with three or more chronic conditions | Rejected in 1^st^ round; unclear relationship between satisfaction with length of consultation and quality |
| Prevalence of multimorbidity | No of pts with three or more chronic conditions | No of pts aged 65 and over | Rejected in 1^st^ round; indicator “Identification of patients with multimorbidity” is given preference, as it is more likely to lead to greater awareness of care needs and the adoption of a clinical approach that takes multimorbidity into account |
| Prevalence of depression | No of pts with depression | No of pts aged 65 and over with three or more chronic conditions | Considered a reference value rather than a quality indicator |
| Prevalence of anxiety disorders | No of pts with anxiety disorder | No of pts aged 65 and over with three or more chronic conditions | Considered a reference value rather than a quality indicator |
| General practitioner coordinated care | No of pts for whom the GP is responsible for care coordination | No of pts aged 65 and over with three or more chronic conditions | Rejected in 1^st^ round; other coordinating health professionals also conceivable option, e.g., care managers |
| Regular check-ups | No of pts having regular check-ups | No of pts aged 65 and over with three or more chronic conditions | Rejected in 1^st^ round; different needs for patients with different combinations of conditions, risk of inadequate care due to rigid scheme |
